# Multifactorial Evaluation of Honey from Pakistan: Essential Minerals, Antioxidant Potential, and Toxic Metal Contamination with Relevance to Human Health Risk

**DOI:** 10.3390/foods14142493

**Published:** 2025-07-16

**Authors:** Sana, Waqar Ahmad, Farooq Anwar, Hammad Ismail, Mujahid Farid, Muhammad Adnan Ayub, Sajjad Hussain Sumrra, Chijioke Emenike, Małgorzata Starowicz, Muhammad Zubair

**Affiliations:** 1 Department of Chemistry, University of Gujrat, Gujrat 50700, Pakistan; sanarajput1216@gmail.com (S.); mianwaqar79@yahoo.com (W.A.); sajjadchemist@uog.edu.pk (S.H.S.); 2 Institute of Chemistry, University of Sargodha, 40100 Sargodha, Pakistan; farooq.anwar@uos.edu.pk; 3 Department of Biochemistry and Biotechnology, University of Gujrat, Gujrat 50700, Pakistan; hammad.ismail@uog.edu.pk; 4 Department of Environmental Sciences, University of Gujrat, Gujrat 50700, Pakistan; mujahid.frid@uog.edu.pk; 5 Department of Chemistry, University of Sahiwal, 57000 Sahiwal, Pakistan; adnanayub@uosahiwal.edu.pk; 6 Department of Plant, Food & Environmental Sciences, Faculty of Agriculture, Dalhousie University, Truro, NS B3H 4R2, Canada; chijioke.emenike@dal.ca; 7 Team of Chemistry and Biodynamics of Food, Institute of Animal Reproduction and Food Research of PAS, 10-748 Olsztyn, Poland

**Keywords:** honey, minerals, antioxidant potential, toxic metals, human health risks

## Abstract

Honey is prized for its nutritional and healing properties, but its quality can be affected by contamination with toxic elements. This study evaluates the nutritional value and health risks of fifteen honey samples from different agro-climatic regions of Pakistan. Physicochemical properties such as color, pH, electrical conductivity, moisture, ash, and solids content were within acceptable ranges. ICP-OES analysis was used to assess six essential minerals and ten toxic metals. Except for slightly elevated boron levels (up to 0.18 mg/kg), all elements were within safe limits, with potassium reaching up to 1018 mg/kg. Human health risk assessments—including Average Daily Dose of Ingestion, Total Hazard Quotient, and Carcinogenic Risk—indicated no carcinogenic threats for adults or children, despite some elevated metal levels. Antioxidant activity, measured through total phenolic content (TPC) and DPPH radical scavenging assays, showed that darker honeys had stronger antioxidant properties. While the overall quality of honey samples was satisfactory, significant variations (*p* ≤ 0.05) were observed across different regions. These differences are attributed to diverse agro-climatic conditions and production sources. The findings highlight the need for continued monitoring to ensure honey safety and nutritional quality.

## 1. Introduction

### 1.1. General Information

Honey, a natural product of *Apis mellifera*, is typically produced through enzymatic activity on nectar [1,2]. According to its origin, honey is divided into monoflora- or multiflora-based natural products [3]. Monofloral honey is derived primarily from the nectar of a single plant species, while multifloral honey originates from the nectar of multiple plant species, reflecting greater botanical diversity. Globally, 1779.6 metric tons of honey is produced, and its market value is expected to increase in the future because of the functional food and nutraceutical potential of this natural product. Worldwide, nearly 28% of honey is produced in China, with Turkey coming in at 5.9%, Iran at 4.5%, the US at 4.1%, and India at 3.5% [2]. The US, Germany, Japan, France, United Kingdom, Italy, and Spain are the top importers of honey, whereas China, New Zealand, Argentina, Germany, Ukraine, India, and Spain are the top exporters [2,4]. About 11,147 tons of honey is consumed domestically in Pakistan, with an amount of 50 g per person, compared to the considerably greater worldwide average of 150 g per person. The honey trade in Pakistan is projected to be worth USD 50 million, including USD 1.8 million in imports, amid the low demand [5].

Honey is a natural product enriched with antioxidants and minerals, and has been frequently used as food throughout the history of humans because of its high nutritional and medicinal value [6,7]. Honey of different origins has different mineral contents, which also reflect the honey’s color [8]. For example, pale honey has a mineral content of about 0.02%, whereas dark honey has a mineral content of about 11.03% [9]. The quality of the honey product and its biochemical properties vary depending on the source of nectar, as different nectar sources produce different types of honey [10,11,12].

The quality of honey is determined through the evaluation of different physicochemical parameters, as set by the International Honey Commission (IHC) [13]. Important quality parameters for the physicochemical characteristics of honey include pH, electrical conductivity (EC), ash and moisture, and color [14]. The floral sources of nectar play a significant role in determining the color, taste, and both the chemical and physical properties of honey. The diversity of pollen and nectar from different plant origins influences the nutrient content and, consequently, the physicochemical characteristics of honey [15]. Honey with a low moisture content does not support microbial growth, whereas higher moisture can promote the growth of yeasts and other microorganisms, leading to sugar fermentation and changes in the honey’s overall taste [16].

### 1.2. Honey in Environmental Monitoring

Honey and honeybees are excellent bioindicators of environmental pollution [17]. The mineral content in honey is a feature that reflects the environmental conditions as well as the geological region of nature’s product [18]. A few of the heavy metals present in honey are essential nutrients but become toxins when in higher amounts [19]. Mineral contents have both toxic and essential metals, and these are beneficial in set standards described by the Ethiopian standard [20], Codex Alimentarius standard [21], and EU standard [22], but concentrations above these thresholds can be harmful to human health [19].

It is observed that due to some anthropogenic activities (usage of pesticides), the honey is contaminated, which may pose health-hazardous effects [23,24]. Several studies have discovered Pb, Cr, Fe, Cu, Ni, and Zn in honeybees, honey samples, and byproducts, all of which are employed as environmental monitors [25,26,27]. Higher concentrations of Pb, Cd, Cr, and Ni are typically linked to the geochemistry of a region or a contaminated environment, which is due to the micro-polluting metals [28,29]. Honey frequently contains trace levels of the metals Zn, Cu, and Mn, which are safe for human health, but in a certain range. However, trace amounts of the hazardous metals Cd, Cr, and Pb may be harmful to our health [29]. Higher concentrations of trace elements, including Pb, Cd, Cu, and Zn, are found in soil due to industrial processes, automobiles, coal ignition, and air pollution from melting and burning municipal waste [25].

### 1.3. Pro-Healthy Properties of Honey

Honey has multiple health benefits that can be linked to its nutraceutical properties, such as antioxidant, antibacterial, and anti-proliferation effects [30]. According to newly conducted research, honey is a significant factor in lowering the risks associated with COVID-19 [31].

Honey, as a rich source of antioxidants, works well against oxidative reactions and can reduce the risk of multiple inflammatory processes, cancer, and cardiac disorders [8]. The antioxidant qualities of various honey varieties vary greatly depending on the floral source [32]. Honey is high in phenolics content, which mainly affects its antioxidant profile [33,34]. It is believed that honey’s natural antioxidants counteract the negative effects of oxidizing agents like oxygen and protect against oxidative stress, and are vital for human health and food preservation [8,32]. Moreover, it is an important natural sweetener product to boost the immune system incredibly [35].

### 1.4. Research Justification

Pakistan is an agricultural country with a wide spectrum of botanical and floral sources that are used as a source of nectar by honeybees. Thus, the production of honey involving diverse floral means coupled with uncontrolled local environmental practices is expected to significantly influence the food quality of this product. Therefore, this study aims to evaluate the physicochemical characteristics and mineral assessment along with the antioxidant potential of different kinds of honey. Quality-oriented physicochemical attributes of honey are investigated and compared with set limits described by the Ethiopian standard [20], Codex Alimentarius standard [21], and EU standard [22]. ICP-OES analysis of the mineral contents of honey provided qualitative and quantitative data on essential and toxic metals, which were compared to the subsequent mineral profiles of other countries and the specific acceptable limits. Human health risk assessments were made for both children and adults associated with toxic metals. The antioxidant potential of the honey was assessed in connection with the nutraceutical prospects of honey. Hence, this study aims to assess the levels of essential minerals, antioxidant capacity, and toxic metal contamination in relation to potential risks to human health.

## 2. Materials and Methods

### 2.1. Samples Selection, Chemical and Reagents

In this study, fifteen (15) raw honey samples with multiflora origin, 250 g of each, were collected from three different areas of Pakistan named Faisalabad (FSD), Azad Kashmir (KSH), and Gujranwala (GRW). Samples were collected from five different locations in each area and then pooled to make one composite sample; a total of three composite samples labelled H-1, H-2, and H-3 were prepared and processed. Samples were stored in airtight plastic jars cleaned with methanol to prevent microbial contamination and brought to the research laboratory, Department of Chemistry, University of Gujrat, for further analyses. All the samples were then stored in the refrigerator at 40 °C till further analysis to prevent the honey from any physicochemical composition changes due to surrounding environmental conditions [36]. The information regarding sample code, floral type, geographical location, and physical appearance/color of honey samples is given in Table 1.

Nitric acid (HNO_3_, 65% m/m) for the preparation of solution for digestion of sample, were purchased from Hajvery Scientific Store, Lahore-Pakistan originate to Merck, Germany. Certified reference material of each metal (1000 mg L−1, Merck, Germany) were employed for instrument calibration after diluting it with HNO_3_ (2% *v*/*v*). DPPH (2,2-diphenyl-1-picrylhydrazyl), BHT (butylated hydroxyl toluene), Folin-Ciocalteau regent, gallic acid, sodium carbonate, H_2_O_2_ and methanol were used of analytical grade purity level and purchased from the Sigma-Aldrich (Germany).

### 2.2. Physicochemical Analysis

#### 2.2.1. Color

According to the Pfund classifier, the intensity of the color of the honey samples was measured. Results were expressed in the ‘Pfund index’. Briefly, the honey samples were diluted with distilled water, and the absorbance of all the samples was measured by a UV-Vis spectrophotometer at 635 nm. After the conversion of absorbance values, color was measured by the Pfund scale [37]. The readings were taken in a triplicate manner for each of the honey samples using approved standards for color analysis by the US Department of Agriculture [38].

#### 2.2.2. pH Value Determination

The pH of honey samples was measured by the pH meter (Adwa AD 1030, Bucharest, Romania). For this purpose, 10 g of the honey sample was taken in a beaker, 50 mL of deionized water was added, and the pH was recorded [39].

#### 2.2.3. Electrical Conductivity

The Electrical Conductivity of honey samples was determined by the conductometer (Adwa AD 3000, Romania). For this test, 10 g of the honey was taken into a beaker, 50 mL of deionized water was added, and conductivity was measured [40].

#### 2.2.4. Moisture and Total Solids Contents

The moisture contents of honey samples were determined by the standard procedure [41]. For this purpose, 5.0 g of honey was taken in a pre-weighed China dish and placed in an oven at 105 °C for 7–8 h. The samples were dried until constant weight was gained, and moisture contents were determined gravimetrically. The moisture and solids contents were calculated by the formula:

(1) $$\%\;Moisture=\frac{loss\;on\;weight}{actual\;weight}\times 100$$

This formula determines the total solids content:

(2) *Total solids* = 100 − *moisture*

#### 2.2.5. Ash Content

The ash contents of honey samples were determined by the standard procedure 925.09 [41]. When the moisture analysis was completed, a known quantity of the sample was placed in a muffle furnace for ash analysis. The sample was placed in the furnace at 600 °C for 4–5 h. Then, after cooling, the China dish was weighed, and the following formula was used to analyze the percentage ash content:

(3) $$\%\;Ash=\frac{weight\;of\;ash}{weight\;of\;sample}\times 100$$

### 2.3. ICP-OES Analysis of Metals

The samples for minerals analysis were prepared according to the method reported by Ahmad et al., Chudzinska et al., and Voyslavov et al. [3,42,43] with some modifications. Precisely 5.0 g of honey was weighed and placed in a microwave digestion vessel, with the addition of 9.0 mL of 65% HNO_3_. The solution was heated until complete digestion was achieved and then allowed to cool at room temperature for an hour. After that, 3.0 mL of H_2_O_2_ was added, and the solution was subjected to continued heating in the oven. After this, the solution was filtered and transferred to a 50 mL flask and the volume made up to the mark with deionized water. The mineral composition was analyzed by an Inductively Coupled Plasma Optical Emission Spectrometer (ICP-OES), iCAP 7400 (Thermo Fisher Scientific, iCAP 7400, US) with a radial view plasma echelle type 52.91 grooves/mm ruled grating. Operational conditions for ICP-OES are summarized in Table 2. Mineral contents, i.e., toxic metals (10) and essential metals (6), were analyzed. After readings, values were expressed in mg/kg. All the samples were analyzed in triplicate, and results are reported as the mean. Calculations were performed with the help of the calibration curve of reference standards.

#### 2.3.1. Human Health Risk Assessment

Health Risk Assessments of ingestion for toxic metals (aluminum, bismuth, cadmium, chromium, copper, manganese, nickel, lead, strontium, and zinc) were calculated. The Average Daily Dose, Total Hazard Quotient and Index, and Carcinogenic Risk Assessment were determined for both children and adults by using Reference dose (RfD) and Carcinogenic Slope Fcator (CSF) values in Table 3.

##### Average Daily Dose

The Average Daily Dose of ingestion (ADDing), in mg kg^−1^ day^−1^, of metals is calculated by using the following formula:

(4) $${ADD}_{ing}=C\times \frac{InhR\times EF\times ED}{BW\times \times AT}\times 10^{-6}$$

where C is the metal’s concentration (mg/kg), the ingestion rate (mg/day) is ‘IngR’ (for children is 200 and for adults, 100), the exposure frequency is ‘EF’ (days/year is 180), the exposure duration is ‘ED’ (for children six (6) years and adults is twenty-four (24) years), the mean body weight is ‘BW’ (15 kg for children and 70 kg for adults), and average time ‘AT’ is 365 × ED.

##### Total Hazard Quotient

The Total Hazard Quotient is the ratio of the Average Daily Dose (mg kg^−1^ BW day^−1^) to the Reference dose (RfD) (mg kg^−1^ BW day^−1^), which is normally used to estimate the potential non-carcinogenic risk of exposure to metals in humans, and is calculated by the following equation:

(5) $$THQ=\frac{{ADD}_{ing}}{RfD}$$

The Hazard Index (HI), which is the sum of all (ingestion) expected HQs, is utilized to count the total potential, i.e., the non-carcinogenic risks of various pollutants.

(6) *HI* = ∑ *HQ* = *HQ_ing_*

If the hazard index is less than or equal to one, then it is deputed as no significant hazard of non-carcinogenic effects, and if it is greater than one, then it might cause adverse health effects.

##### Carcinogenic Risk Assessment

The danger of cancer for LCR (Lifetime Cancer Risk) of metals was assessed to find the health danger or hazard from cumulative life cancer risk. It was calculated by following the formula:

(7) *LCR* = *ADD_ing_* × *CSF*

(8) *LCR* = ∑ *Cancer Risk* = *Cancer Risking*

### 2.4. Antioxidant Activity Evaluation

#### 2.4.1. Total Phenolics Content

All samples of honey were diluted by adding 0.1 g of honey in 50 mL of distilled water. Different concentrations of 200, 400, 800, and 1000 ppm were prepared. An amount of 1.0 mL of each sample was taken in separate test tubes, and then 5 mL of 10% Folin–Ciocalteu reagent was added as described earlier by Singleton et al. [44] with some modification. Solutions were then placed in an incubator for 5 min at room temperature. Then 4.0 mL of 7.5% Na_2_CO_3_ was added, and the solution was left to stand for 2 h till the reaction was completed. In this assay, a chemical reaction occurred between Folin reagent and the phenolic compounds (which were present in the sample), forming a blue color complex that absorbs calorimetric radiation allowing the overall quantification of these compounds [45]. Then, the value of absorbance for each sample solution was measured at 760 nm using a UV-Vis spectrophotometer (C-7200 from PEAK Instruments (Shanghai, China). Calculations were made using the standard calibration curve (concentration vs. absorbance). The result was then expressed in Gallic acid equivalent (GAE).

#### 2.4.2. DPPH Radical Scavenging Assay

The samples of honey were diluted by adding 0.1 g of honey in 50 mL of distilled water. Different concentrations such as 200, 400, 800, and 1000 ppm were prepared. A modified DPPH radical scavenging assay was employed to determine this antioxidant attribute described by Chotimarkorn et al. [46] with some modification. Briefly, a small portion (1.0 mL) of each sample was taken in a separate Pyrex test tube. Then, 4.0 mL of methanol, along with 1.0 mL of methanolic DPPH solution, were added. Then, all the samples were kept at ambient conditions for the reaction’s completion, and absorbance was noted at wavelength 515 nm by using a UV-Vis spectrophotometer (C-7200 from PEAK Instruments (China)). In the DPPH assay, unpaired e- is present, which is used to form a pair with H+ donated by free radical scavenging antioxidants, which are present in the samples. By this reaction, the DPPH color changes from purple to yellow, and this degree of decolorization is measured by a UV-Vis spectrophotometer at 515 nm [47]. Butylated hydroxyl toluene (BHT) was used as a positive control for comparison purposes. The % inhibition was calculated as below:

(9) $$\%\;Inhibition=\frac{Abs.\;of\;control-Abs.\;of\;sample}{Abs.\;of\;control}\times 100$$

### 2.5. Statistical Analysis

All assessments were carried out in triplicate and the data were computed as mean ± standard deviation for triplicate experiments. Various statistical operations were applied to the data. Pearson correlation and Principal Component Analysis (PCA) were performed on both mineral and physicochemical analysis. Data analysis was performed by using MS Excel software (version 1.5) for basic data analysis of mean and standard deviation, GraphPad Prism (version 10.0.0), R software (version 4.4.2) for PCA, heat map, and SPSS (version 26) for correlation.

## 3. Results and Discussion

### 3.1. Physicochemical Parameters

The data for the physicochemical characteristics of composite H-1, H-2, and H-3 honey samples is presented in Table 4. The quality of honey, its season of production, time of storage, nectar source, water contents, and botanical origin significantly affect its physiochemical properties [48]. The season of harvesting, storage conditions, as well as the degree of maturity of honey in the hive, may affect the physicochemical attributes of honey [48]. In the current work, moisture content was assessed with a maximum mean value of 17.07 ± 1.42%, which is higher than the 16.00 ± 2.19% reported for samples of honey from Nigeria by Buba et al. [33]. The moisture contents of the samples analyzed were within the limit of international quality standards. A low moisture contents did not allow the survival of many of the microorganisms such as bacteria and yeast, whereas a higher moisture level can promote the survival of many such entities [16]. Reciprocating moisture, the total solids of honey were evaluated with a maximum value of 82.93 ± 1.42. Another important parameter of honey is ash content, which represents the inorganic composition of honey and classifies very important features of honey [49]. The ash content is evaluated to be 0.10 ± 0.06%, which is quite a lot lower than the ash content (0.59 ± 0.03%) reported by Fahim et al. in Pakistan [50]. Such a variation can be understandable as the nectar ingredients could directly affect the ash content in honey samples [51].

An important physical parameter of honey is pH, which is also recorded for the honey sample, depicting overall an acidic range up to 5.02 ± 0.19. The recorded pH value was higher than the mean value of 4.19 ± 0.7 reported earlier in a related study from Pakistan by Fahim et al. [50]. A low pH value indicates that organic acids such as pyruvic, malic, gluconic, and citric acid may be present in the honey sample [50]. It is evident from the findings of the reported study by Aljohar et al. that naturally produced honey is acidic in nature with a pH value ranging from 3.42 to 6.10 [52]. The pH of composite H-1 honey was more acidic at a value of 4.87 ± 0.32, while the composite H-3 sample had a less acidic nature with a pH value at 5.23 ± 0.33, whereas the pH for composite H-2 was at 4.97 ± 0.31 [53]. The electric conductivity (EC) value is typically correlated with the total ionic content of the material, and in the honey, it was observed to be in the range of 0.1–3 mS/cm [54]. The values of electrical conductivity for all the tested samples of honey were within acceptable standard limits with an overall value as high as 0.70 ± 0.07. However, this EC value is slightly lower than that reported earlier (0.89 ± 0.02 mS/cm) by Nemo et al. [53].

Color is an important parameter for the categorization of honey multiflora. The color of honey ranges from light yellow to dark amber, and in extreme cases black and maybe green or red [55]. The colors of all the samples of honey collected from different locations and regions were observed as ranging from extra light amber to almost transparent. The lightest color value was evaluated as 27.31 ± 4.90 mm for H-2 and dark amber with 59.23 ± 4.71 mm for the H-1 sample. The Pfund values recorded in our study were lower than those reported for Saudi honey (113.82 ± 2.19), Egyptian honey (73.88 ± 2.29), and Kashmiri honey (89.45 ± 1.17) [15]. In another research, Boussaid et al. [56] found values in the range of 36.34–51.37, quite close to our data. Variations in color values can be attributed to different flora and geographical origins [53,57,58]. The honey samples (H-1, H-2, and H-3) showed better quality compared with reference quality standards. These values are compared with the standard range values given by the Ethiopian standard [20], Codex Alimentarius standard [21], and EU standard [22].

The results of the Pearson correlation (r) between physiochemical characteristics are shown in Table 5. The color showed a highly positive significant correlation to moisture content (r = 0.954808); this suggests that the degree of honey’s color increases substantially with its increasing moisture level. However, the analysis revealed a moderate positive correlation (r = 0.618109) between color analysis and ash content. Pearson correlation showed a strong relationship between ash and moisture content (r = 0.823821), which implies that more moisture in honey is linked to a higher mineral (ash) concentration. The correlations of electrical conductivity to pH (r = 0.891707), honey’s mineral content, and ionic makeup are reflected in this relationship. There was a strong negative correlation of color with total solids (r = −0.95481) and EC with ash (r = −0.99805). The relationships show the interdependence between honey’s chemical characteristics (moisture, ash, pH, EC) and physical characteristics (color). Color and moisture and EC and pH have significant correlations, suggesting that these factors may be used as markers of honey quality, authenticity, or botanical provenance.

**Figure 1:**
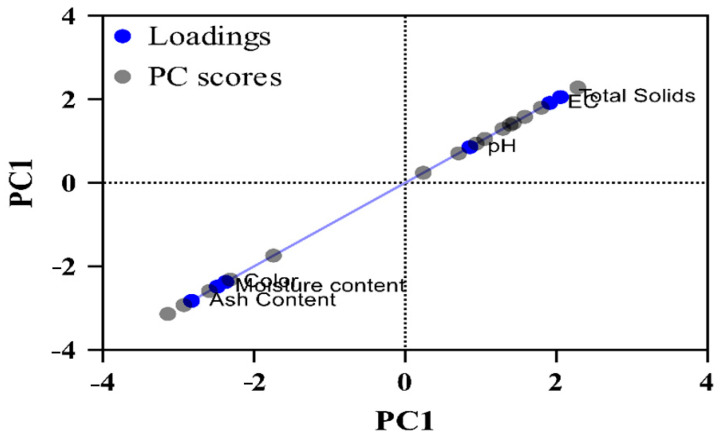
Biplot of PCA analysis of physicochemical characteristics of honey samples from different agro-climatic regions.

The physicochemical characteristics of honey samples from different agro-climatic regions are represented in the form of Principal Component Analysis (PCA), i.e., biplot (Figure 1), eigenvalues, and proportion of variance. PC1 showed the highest eigenvalue, i.e., 3.77 and had more significant variation in data followed by PC2 (1.31), PC3 (0.516), PC4 (0.374), and PC5 (0.0288). PC1 and PC2 explain most of the variability in data. Five principal components contribute to its variance, 62.81%, 21.88%, 8.59%, 6.24%, and 0.48%, respectively. The contribution to the cumulative proportion of variance by five PCs is 62.81%, 84.89%, 93.28%, 99.52, and 100%, respectively. The data represents that PC3, PC4, and PC5 have minor variations and represent less significant features in the data.

### 3.2. Mineral Analysis

Trace elements, including Fe, Zn, Cu, Mn, Cr, and Ni, which humans need in amounts of less than 50 mg/day, and major elements like K, Ca, and Mg, which are required at >50 mg/day, were determined, while Al, Ba, Bi, B, Cd, and Pb are ultra-trace elements that frequently exist in the dry matter of the routine diet at levels less than 50 ng/day and typically less than 1.0 µg/day [59,60]. Variability in the content of major elements such as potassium in foods is important because potassium plays a critical role in maintaining normal cellular function, nerve transmission, and muscle contraction, all of which are essential for overall health and physiological balance [61]. Insufficient potassium intake is associated with increased risk of hypertension and cardiovascular diseases [62]. Mineral analysis of honey samples H-1, H-2, and H-3, representing the concentrations of metals (both essential and toxic metals), is given in Table 6 and Table 7. Studies report that there are various means of mineral enrichment in honey, in particular, the minerals present in the soil that dissolve in water and are taken up by plant roots and shifted to plant nectar via an osmoregulatory process. Such soil-derived minerals and metals are transferred through honeybees while interacting with nectar [63].

#### 3.2.1. Essential Minerals

**Figure 2:**
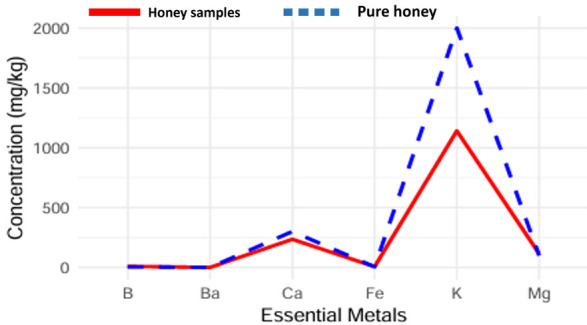
A comparative presentation of minerals in honey samples and pure honey.

The most abundant essential mineral detected in honey samples was potassium (K), ranging from 922 to 1136 mg/kg (average level of 1018 mg/kg), followed by calcium (Ca), magnesium (Mg), iron (Fe), boron (B), and barium (Ba) as depicted in Table 6. A comparison of the essential minerals from a pure honey source and the honey samples of three different regions reveals that almost all minerals are similar in concentrations except potassium (K) with lesser concentrations; Figure 2. This variation may be due to the climatic conditions, the nature of the soil, and the low K in the water of the region.

In the present research, the potassium value is higher than the mean reference value of 135 mg/kg reported in Ethiopian honey by Gebeyehu et al. [64] but lower than the mean value (2250.39 mg/kg) of honey from Poland reported by Tarapatskyy et al. [65]. Calcium is the second most prevalent mineral, with a mean value of 124 mg/kg. The calcium content of honey in the present analysis is higher than the mean value of 85.2 mg/kg reported by Hungerford et al. in Australian honey [24]. According to other studies, both of these essential minerals are the most significant nutrients in honey [63,64]. The maximum value of potassium was found in the honey sample H-1 and the minimum in H-2. Barium was noted to have a minimum level, less than 0.01 mg/kg, which was lower than the mean value of 0.3 ± 0.2 mg/kg reported earlier by Hungerford et al. [24]. Subsequently, B, Fe, and Mg were found to be at 5.49 ± 2.88, 3.88 ± 2.33, and 68.77 ± 41.41 mg/kg, respectively. The mean content of boron (B) in the present study is slightly higher than that reported earlier (4.7 ± 2.2 mg/kg) by Hungerford et al. [24], while the present mean level of iron in honey is quite close to that of an earlier study (3.80 mg/kg) reported by Akharaiyi et al. [48], and the mean content of Mg is lower than that reported earlier (80.70–119.30 mg/kg) by Alqarni et al. [66]. Magnesium contents were above the limit set by the Codex standard, i.e., 25 mg/kg, whereas iron concentrations were under the tolerable standard set by the Codex standard, i.e., 15 mg/kg [14].

**Figure 3:**
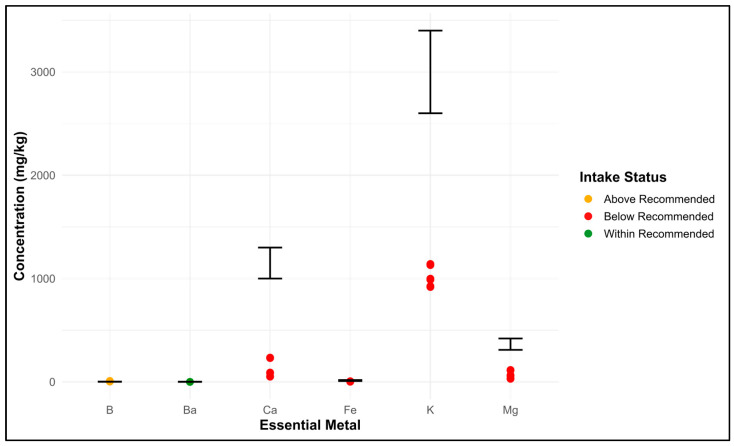
Essential minerals above, below, and within the limit in honey samples.

Figure 3 shows the comparative relationship between the individual sample’s essential minerals and the recommended daily intake value (RDI) for health taken from the literature. Green, red, and orange dots represent values within the recommended limit, below the limit, and above the limit, respectively. In terms of assessing nutritional value and health risks, the error bars provide a clear and insightful comparison by representing scientifically defined intake levels. Black lines indicate the RDI. In the present study, boron was found to be beyond the limit, barium within the limit, and potassium, calcium, iron, and magnesium were found to be below the recommended standard levels. These findings suggest that honey can serve as a source for the mineral shown by the green dot, i.e., barium. However, it cannot be considered as a significant dietary source for Ca, Fe, K, and Mg. In the case of B, monitoring is important due to its narrow safety margin.

The Pearson correlation (r) of essential metals in honey samples is shown in Table 7. There was a highly significant positive correlation between Fe and B (r = 0.94397), so a significant positive correlation indicates that the amounts of boron and iron in honey are strongly correlated. Larger levels of boron are typically linked to larger levels of iron. A strong positive correlation between Ca and B and between Ca and Fe (r = 0.999836 and r = 0.949787) indicates that the Ca level depends on B and Fe. The correlation of Mg to B, Fe, and Ca, with respective r values (r = 0.980388, r = 0.990498, and r = 0.983794), indicates a strong relationship between magnesium and calcium, boron, and iron, indicating that these metals may have comparable origins or external factors. The positive correlation between K and B, Fe, Ca, and Mg (r = 0.985713, r = 0.986072, r = 0.988599, and r = 0.999576) suggests that these metals have a common route or a robust geochemical association. There is a moderate negative correlation between Fe and Ba (r = −0.48488), suggesting that distinct components in the honey matrix have different origins, levels of movement, or interactions.

#### 3.2.2. Toxic Metals

The abundant toxic metal determined in honey samples was aluminum (Al), ranging from 4.13 ± 0.11 to 9.08 ± 0.32 mg/kg with an average content of 6.11 ± 2.62 mg/kg (Table 8). A recent study shows a higher mean content of Al (1.2 mg/kg) than in Australian honey [24]. The higher value of aluminum was noted in H-1 (9.08 ± 0.32 mg/kg) and the minimum in the H-2 (4.13 ± 0.11 mg/kg) honey sample.

Cadmium and nickel were determined with contents less than 0.01 mg/kg, which is below the quality reference standards. The safe limit for nickel (Ni) set by the FAO/WHO expert committee on food additives (ECFD) is 5.0 mg/kg [14]. The standard limit for cadmium (Cd) is 0.05 mg/kg according to European legislation and the Codex [21]. According to the Codex standard, the threshold limit for zinc (Zn) and copper (Cu) is below 5.0 mg/kg [21]. In the present study these metal levels were found to be below the permissible limits.

In the present study, the Cu value was higher than the mean value of 0.05 ± 0.02 mg/kg reported earlier by Nemo et al. [53]. Lead (Pb) was found with an overall average concentration of 0.18 ± 0.05 mg/kg, which was below the standard limit of 3.0 mg/kg suggested by FAO/WHO [67]. The present study’s lead value is higher than the mean value of 0.028 ± 0.074 mg/kg reported by Hungerford et al. [24]. However, Cd and Pb are considered bioindicators for contamination in honey [68]. Pb is a toxic metal and has adverse and long-lasting effects on children and causes high blood pressure and kidney damage in adults [69]. The present study’s mean Cd level is close to that reported in the study by Hungerford et al. [24]. Among heavy metals, Pb is the most hazardous substance, which is commonly introduced into the environment through vehicular emissions, whereas Cd originates from industrial waste incinerators moving from soil to plant; both metals directly contaminate the nectar [70]. The concentrations of Bi, Cr, Mn, and Sr were noted with overall values of 0.08 ± 0.04, 0.54 ± 0.02, 0.59 ± 0.12, and 1.14 ± 1.38 mg/kg, respectively. In the current analysis, Mn, Zn, and Ni levels are lower than the ranges 4.15–6.04, 3.44–5.72, and 0.15–0.67 mg/kg, respectively, reported in the literature by Alqarni et al. [66]. The average values of Sr and Cr in the present study are higher than the mean values of 0.4 ± 0.3 and 0.008 mg/kg observed earlier by Hungerford et al. [24]. The observed Mn and Cr levels are under the limit set by WHO, i.e., 5.5 µg/g and 100 µg/g, respectively [28,71]. Nevertheless, at high levels, these metals negatively impact the operation of several organs and cell functions [72].

The results of the Pearson correlation (r) of toxic metals in honey samples are shown in Table 9. According to the correlation, there was a perfect negative correlation (r = −1) between Ni and Cd. According to this association, it is questionable that these two metals will coexist in the same samples at high concentrations. This could be because of different environmental sources or competitive exclusions in soil or plants. Moreover, a highly significant positive correlation was found between Cr and Cd (r = 0.998337), Mn and Al (r = 0.959199), Ni and Mn (r = 0.908928), Pb and Ni (r = 0.980245), Sr and Al (r = 0.997496), Sr and Mn (r = 0.976791), and Zn and Cu (r = 0.990897). These significant positive correlations indicate that metals are closely related and share environmental, anthropogenic, geochemical, industrial, and agricultural sources. However, a high significant negative correlation occurred between Cu and Bi (r = −0.99329), Mn and Cd (r = −0.90893), Ni and Cr (r = −0.99834), Pb and Cd and Cr (r = −0.98025 and r = −0.99002), and Zn and Bi (r = −0.99982), which suggested that metals might have originated from different processes and sources due to competing bioaccumulation or environmental interactions.

The present analysis revealed a moderate positive correlation between Bi and Al (r = 0.713026), Ni and Al (r = 0.753956), and Sr to Ni (r = 0.798523), while indicating a moderate negative correlation between Cd and Al (r = −0.75396), Cr and Al (r = −0.71484), Cu and Al (r = −0.78934), Sr and Cd, Cr, and Cu (r = −0.79852, r = −0.7625, and r = −0.74395). The health hazards of toxic metals such as chromium (Cr), lead (Pb), and cadmium (Cd) have been widely accepted, necessitating their reliable estimation in food products such as honey. The main sources of hazardous metal contamination in foods mainly include the increasing magnitude of environmental pollution caused by rapid industrialization and automobile expansion, as well as diverse anthropogenic activities. The high positive connection between Cr and Cd suggests that these harmful metals are commonly found in the environment. Negative correlations may indicate regions with higher concentrations of one harmful metal than another, which could help guide mitigation measures.

**Figure 4:**
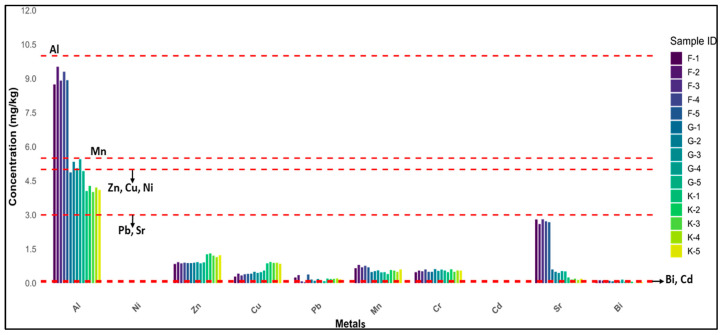
Concentration vs. regulatory limits of toxic metals in honey samples. Samples’ codes according to the origin area: Faisalabad (Punjab, F1–F5): Chak 116 JB, Chak 436 GB, Chak 102 RB, Chak o1 JB, Chak 65 RB; Kashmir (Pakistan, K1–K5): Barnela, Cham, Kulian, Kote Jamel, Bhimber; Gujranwala (Punjab, G1–G5): Kamoki, Kalaske, Lohianwala, Nendipur, Aroop.

In Figure 4 every bar shows the presence of toxic metals in the individual honey samples selected for this research. The plot shows the relationship between the toxic metal concentration of all individual samples and their regulatory limits. The red dashed lines represent the safe limits of each metal. A possible health hazard could arise if the metal concentration rises above the acceptable threshold. If the bar crosses the dashed line, it exceeds the safe limit, and it may pose serious potential risk associated with the specified metal. Analysis revealed that all the honey samples are safe concerning the regulation limits of metals set by international standards.

**Figure 5:**
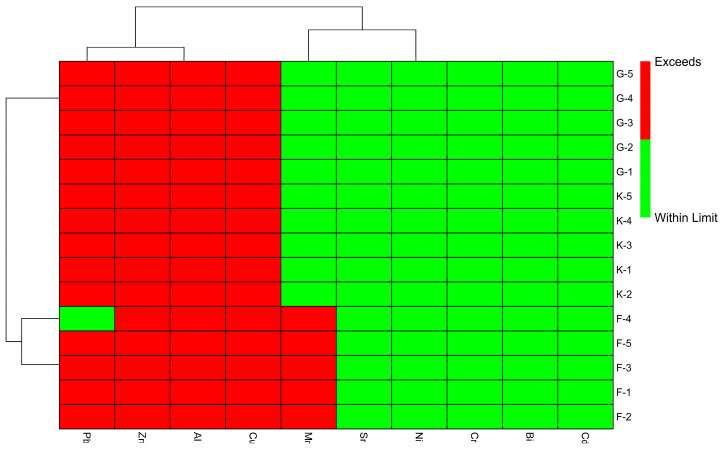
Heat map of toxic metal exceedance in honey samples. Samples’ codes according to the origin area: Faisalabad (Punjab, F1–F5): Chak 116 JB, Chak 436 GB, Chak 102 RB, Chak 01 JB, Chak 65 RB; Kashmir (Pakistan, K1–K5): Barnela, Cham, Kulian, Kote Jamel, Bhimber; Gujranwala (Punjab, G1–G5): Kamoki, Kalaske, Lohianwala, Nendipur, Aroop.

Figure 5 is a heat map representing the exceedance status of metals in individual samples with respect to RfD values, whether they meet the safe limit or not. The green color blocks show the safe limits with zero concentration, whereas the red color blocks show the concentration above the safe limits. According to the heat map, the maximum concentration limit is 1, and concentrations of the metals exceeding 50% of the level are a risk for health, whereas those below are safer. Considering the two-way presentation of the heat map, it was observed that the sample from Faisalabad (F-1–F-5) exceeded the safe limit and has a risk with regard to Mn, Cu, Al, Zn, and Pb (the Pb level was safe in F-4), whereas the samples from Gujranwala (G-1–G-5) and Kashmir (K-1–K-5) exceeded the safe limit and have a risk with regard to Cu, Al, Zn, and Pb. However, the findings suggest that the level of Mn was safe in all Gujranwala and Kashmir samples. The findings suggest that all the samples from Gujranwala, Kashmir, and Faisalabad had high concentrations of zinc, but zinc is not as harmful as other heavy metals because it is necessary for human health. However, an optimized use of honey can be healthier for humans without health risks. It is also observed through the heat map that all the honey is safe in terms of Cd, Bi, Cr, Ni, and Sr metals.

**Figure 6:**
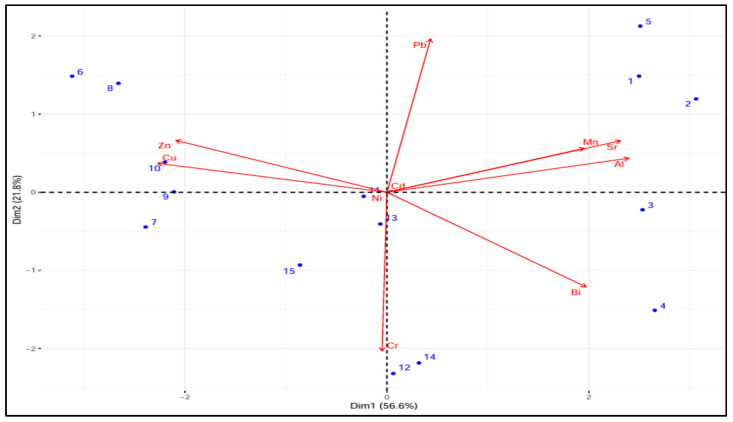
PCA biplot of toxic metals. Samples’ codes according to the origin area: Faisalab (Panjeb, F1-F5): 1—Chak 116 JB, 2—Chak 436 GB, 3—Chak 102 RB, 4—Chak 01 JB, 5—Chak 65 RB; Kashmir (Pakistan, K1-K5): 6—Barnela, 7—Cham, 8—Kulian, 9—Kote Jamel, 10—Bhimber; Gujranwala (Punjab, G1-G5): 11—Kamoki, 12—Kalaske, 13—Lohianwala, 14—Nendipur, 15—Aroop.

A biplot of toxic metals for individual samples is represented in Figure 6. Al, Cr, and Zn dominate PC1 (60–70% variance), suggesting that they are the main contributors to sample differentiation. Mn and Sr have an impact on PC2 (20–30% variance), indicating a secondary source of variation. Cu and Zn confirm their association by pointing in the same direction. The close clustering of samples F-1 through F-5 suggests that their metal profiles are comparable. Another group, K-1 to K-5 and G-1 to G-5, represents different metal compositions. PCA verifies that metals such as Al, Cr, and Zn are the main causes of variation, with Mn and Sr playing a secondary role (Figure 6).

##### Health Risk Assessment of Toxic Metals

Health Risk Assessment profiles of honey samples were assessed for Al, Bi, Cd, Cr, Cu, Mn, Ni, Pb, Sr, and Zn (TMs), including the Average Daily Dose for ingestion, Total Hazard Quotient and Index, and Carcinogenic Risk Assessment determined for both children and adults. Using values of the Reference dose (RfD) Carcinogenic Slope Factor (CSF) of metals from Table 3, the risk assessment model of all the honey samples was evaluated by formulas and is represented in the form of Table 10. In terms of the ADD_ing_ of honey, children received the highest dose of all metals (ADD_ing_ = 5.34 × 10^−8^ − 6.06 × 10^−5^ for H-1, 5.34 × 10^−8^ − 2.75 × 10^−5^ for H-2 and 5.34 × 10^−8^ − 3.42 × 10^−5^ for H-3), whereas adults received (ADD_ing_ = 5.60 × 10^−9^ − 6.36 × 10^−6^ for H-1, 5.6 × 10^−9^ − 2.89 × 10^−6^ for H-2 and 5.6 × 10^−9^ − 3.59 × 10^−6^ for H-3).

This implies that children’s smaller body weight and comparatively higher intake per unit of body mass expose them to metals in honey at higher levels. Values that are close to or higher than the reference doses (RfDs) set by regulatory agencies (such as the EPA or WHO) may be harmful to health over an extended period. Furthermore, all metal HI values in soil were determined to be less than 1, suggesting no significant risks to human health. If the hazard index is less than or equal to one, then it is assigned as no significant hazard of non-carcinogenic effects, and if it is greater than one, then it might cause adverse health effects, indicating a discernible health risk.

Lifetime exposure to toxic metals was assessed through the carcinogenic slope factor. An LCR value < 1 × 10^−4^ exhibits no risk of cancer in humans. The assessment revealed that none of the toxic metals exhibited carcinogenic risk. In terms of the LCR of honey, children received a high dose of all metals (LCR = 9.09 × 10^−5^ for H-1, 5.06 × 10^−5^ for H-2 and 5.53 × 10^−5^ for H-3), whereas adults received (LCR = 9.54 × 10^−6^ for H-1, 5.31 × 10^−6^ for H-2 and 5.81 × 10^−6^ for H-3). Thus, in the current study area, children are more vulnerable than adults. Metal exposure through honey is regarded as a minimal risk if results are below the safe international standard. If they are above safe thresholds, it suggests possible health issues that need to be addressed.

### 3.3. Effect on Antioxidant Activity

#### 3.3.1. Total Phenolic Contents

Total phenolic contents (TPCs) are an important indicator towards assessing the level of total phenols and antioxidant potential of plant foods, including honey, similarly to natural products [73]. The TPCs of tested honey samples were determined by the modified Folin–Ciocalteu method [44] using a UV-vis spectrophotometer at wavelength 760 nm. TPCs were found to be 4.52 ± 2.01, 4.07 ± 3.11, and 4.11 ± 2.10 in g GAE/kg for H-1, H-2, and H-3 samples, respectively. In a reported study by Naqvi et al., TPC values in some analyzed honey samples were observed to be 7.55 ± 0.06 and 3.59 ± 0.04 in g GAE/kg [74], while another research revealed TPCs in 16 floral honey samples to be in the range of 60.5–1008 g GAE/kg by Chang et al. [75]. The TPC of Mexican honey ranged between 79.3 and 5336 g GAE/kg, as recorded by Rodríguez et al. [76]. In the study reported by Aker et al., a Turkish pine honey sample contained 166 ± 5.80 of TPC, while flower honey had 106.04 ± 9.55 g GAE/kg [77].

#### 3.3.2. DPPH Radical Scavenging Activity

**Figure 7:**
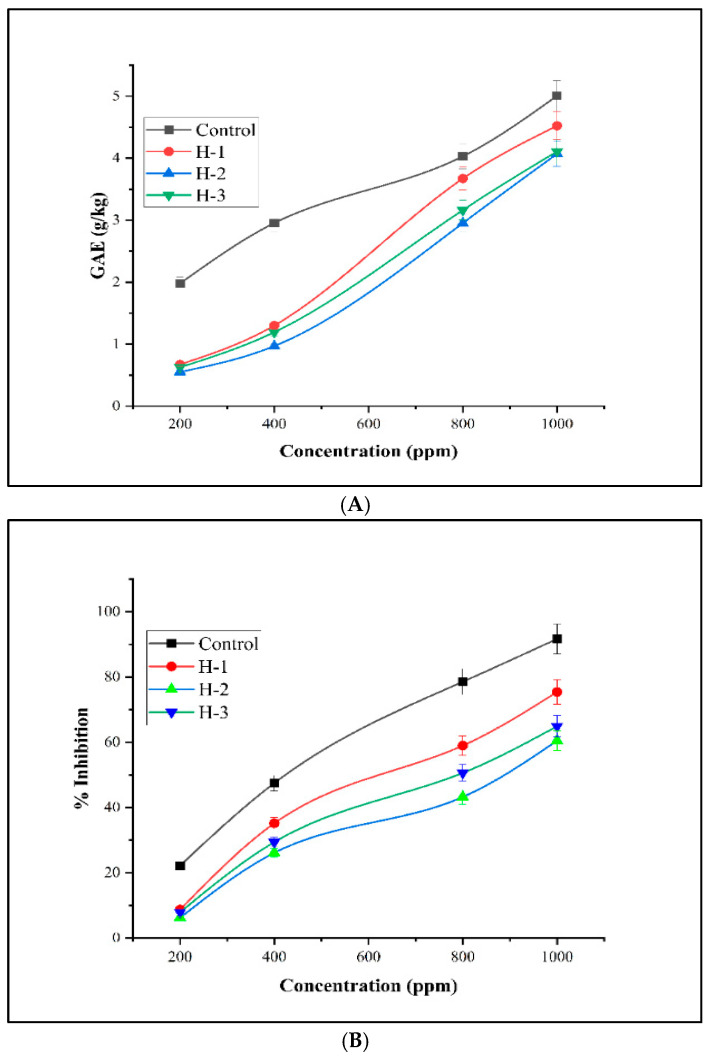
Antioxidant potential. (**A**) Total phenolic contents. (**B**) DPPH % inhibition assay. H-1: honey samples from the region of Faisalabad, Pakistan; H-2: honey from the region of Kashmir, Pakistan; H-3: honey from the region of Gujranwala, Punjab.

The DPPH radical scavenging activity of the tested honey samples is given in Figure 7. The percentage radical scavenging values for H-1, H-2, and H-3 were determined to be 17.83–75.43%, 16.11–60.48%, and 16.93–64.91%, respectively, which were higher than the values reported in the literature by Naqvi et al. [74] in Pakistan. The % inhibition (RSA assay) of honey samples from Korea in a study by Kim et al. was reported as 45% [78]. In China, honey samples were assessed by Sun et al. for assay, which inhibited 50% of DPPH free radicals [79].

## 4. Conclusions

Honey samples were collected from three regions of Pakistan and analyzed for physicochemical properties, antioxidant potential, and mineral (essential and toxic) content, with results compared to international quality standards. Physicochemical analysis confirmed good quality honey, with key parameters such as moisture, color, taste, and pH within acceptable ranges.

Mineral composition varied depending on floral origin, geography, and environmental conditions. High levels of essential minerals such as potassium, calcium, magnesium, zinc, and iron indicated that honey may contribute to dietary mineral intake, particularly in regions with deficiencies. Most essential minerals were within safe limits, except boron, which slightly exceeded the recommended threshold.

Some toxic metals (Mn, Cu, Al, and Pb), likely introduced through environmental pollution and human activity, were found in elevated levels in samples of Gujranwala, Kashmir, and Faisalabad. Although health risk assessments showed no immediate danger, some samples exceeded the permissible limits set by regulatory bodies (e.g., WHO, FAO, EU), highlighting the need for stricter quality control.

Antioxidant analysis showed that honey has strong free radical scavenging capacity, supporting its role in combating oxidative stress. Darker honey samples, in particular, demonstrated higher antioxidant activity, likely due to their greater total phenolic content (TPC). Honey remains a valuable natural product with notable nutritional and medicinal benefits. Future studies should include meta-analyses of existing data and longitudinal assessments across different regions and seasons. Additionally, promoting organic farming and sustainable apiculture, alongside continuous monitoring and stronger regulations, can further enhance honey quality and safety.

## Figures and Tables

**Table 1:** Sample code, floral type, geographical location, physical appearance/color of samples.

Sample	Floral Type	Geographical Origin	Area of Production	Color
F-1	Multiflora	Faisalabad, Punjab	Chak 116 JB	Light Amber
F-2			Chak 436 GB	Light Amber
F-3			Chak 102 RB	Light Amber
F-4			Chak 01 JB	Light Amber
F-5			Chak 65 RB	Light Amber
H-1 Code for all the samples of Faisalabad, Punjab (n = 5)				
K-1	Multiflora	Kashmir, Pakistan	Barnala	Transparent
K-2			Cham	Transparent
K-3			Kulian	Transparent
K-4			Kote Jamel	Transparent
K-5			Bhimber	Transparent
H-2 Code for all the samples of Kashmir, Pakistan (n = 5)				
G-1	Multiflora	Gujranwala, Punjab	Kamoki	Extra Light Amber
G-2			Kalaske	Light Amber
G-3			Lohianwala	Extra Light Amber
G-4			Nandipur	Extra Light Amber
G-5			Aroop	Extra Light Amber
H-3 Code for all the samples of Gujranwala, Punjab (n = 5)				

**Table 2:** ICP-OES Operation parameters.

ICP-OES	Operational Parameters
RF power	1250 W
Plasma coolant gas flow	15 L min^−1^
Auxiliary gas flow rate	1 L min^−1^
Nebulizer gas flow	0.65 L min^−1^
Nebulizer type	V-groove, pressure 240 kPa
Sample uptake rate	1.5 mL min^−1^
Pump rate	35 rpm during flushing and 20 rpm during analysis

**Table 3:** Reference dose (RfD) Carcinogenic Slope Factor (CSF) of metals for ingestion.

Metal	RfD (mg/kg/day)	CSF (mg/kg/day)^−1^
Al	1	1.4
Ni	0.02	1.7
Cd	0.001	0.38
Cr	1.5	0.5
Cu	0.04	1.7
Pb	0.0035	0.0085
Mn	0.14	N/A
Bi	0.2	N/A
Sr	0.6	N/A
Zn	0.3	N/A

N/A, Not Available.

**Table 4:** Physiochemical characteristics (g/100 g) of honey samples in comparison with standards.

Parameters	H-1	H-2	H-3	Mean ± SD	Ethiopian Standard	Codex Alimentarius Standard	EU Standard
Moisture %	18.6 ± 0.83	15.8 ± 0.43	16.8 ± 0.5	17.07 ±1.42	<20	≤21	<20
Ash %	0.17 ± 0.01	0.08 ± 0.01	0.05 ± 0.02	0.10 ± 0.06	<0.6	<0.6	<0.6
Total Solids %	81.4 ± 0.83	84.2 ± 0.43	83.2 ± 0.50	82.93 ± 1.42	-	-	-
pH	4.87 ± 0.32	4.97 ± 0.31	5.23 ± 0.33	5.02 ± 0.19	-	-	-
Color	59.23 ± 4.71	27.31 ± 4.90	47.26 ± 3.61	44.60 ± 16.13	-	-	-
EC	0.63 ± 0.08	0.72 ± 0.03	0.76 ± 0.04	0.70 ± 0.07	<0.8	<0.8	<0.8

H-1: honey samples from the region of Faisalabad, Pakistan; H-2: honey from the region of Kashmir, Pakistan; H-3: honey from the region of Gujranwala, Punjab.

**Table 5:** Pearson correlation of physiochemical parameters of honey.

Physiochemical Parameters	Moisture Content (g/100 g)	Ash Content (g/100 g)	Total Solids (g/100 g)	pH	Color (mm Pfund)	EC (mS/cm)
Moisture Content	1					
Ash Content	0.823821	1				
Total Solids	−1	−0.82382	1			
pH	−0.42223	−0.86168	0.422229	1		
Color (mm Pfund)	0.954808	0.618109	−0.95481	−0.13372	1	
EC	−0.78679	−0.99805	0.786793	0.891707	−0.56778	1

**Table 6:** Essential mineral concentration (mg/kg) in honeys of various agro-climatic regions.

Essentials Minerals	H-1 (mg/kg)	H-2 (mg/kg)	H-3 (mg/kg)	Mean ± SD
Boron (B)	8.77 ± 0.04	3.36 ± 0.19	4.34 ± 0.32	5.49 ± 2.88
Barium (Ba)	<0.01	<0.01	<0.01	<0.01
Iron (Fe)	6.23 ± 0.04	1.57 ± 0.03	3.83 ± 0.06	3.88 ± 2.33
Calcium (Ca)	232.17 ± 1.85	51.77 ± 1.20	87.48 ± 2.20	123.81 ± 95.53
Magnesium (Mg)	113.39 ± 1.67	31.56 ± 1.41	61.37 ± 2.43	68.77 ± 41.41
Potassium (K)	1136.40 ± 4.33	922.18 ± 3.54	994.67 ± 4.97	1017.75 ± 108.96

H-1: honey samples from the region of Faisalabad, Pakistan; H-2: honey from the region of Kashmir, Pakistan; H-3: honey from the region of Gujranwala, Punjab.

**Table 7:** Pearson correlation of essential metals in honey samples.

Metals	B	Ba	Fe	Ca	Mg	K
Boron (B)	1					
Barium (Ba)	−0.169074	1				
Iron (Fe)	0.94397	−0.48488	1			
Calcium (Ca)	0.999836	−0.18688	0.949787	1		
Magnesium (Mg)	0.980388	−0.36	0.990498	0.983794	1	
Potassium (K)	0.985713	0.33267	0.986072	0.988599	0.999576	1

**Table 8:** Toxic metal concentration (mg/kg) in honey samples of various agro-climatic regions.

Toxics	H-1 (mg/kg)	H-2 (mg/kg)	H-3 (mg/kg)	Mean ± SD
Aluminum (Al)	9.08 ± 0.32	4.13 ± 0.11	5.13 ± 0.25	6.11 ± 2.62
Bismuth (Bi)	0.11 ± 0.02	0.04 ± 0.03	0.10 ± 0.04	0.08 ± 0.04
Cadmium (Cd)	<0.01	<0.01	<0.01	<0.01
Chromium (Cr)	0.53 ± 0.05	0.54 ± 0.05	0.56 ± 0.05	0.54 ± 0.02
Copper (Cu)	0.37 ± 0.05	0.89 ± 0.03	0.48 ± 0.05	0.58 ± 0.27
Manganese (Mn)	0.72 ± 0.06	0.53 ± 0.08	0.51 ± 0.04	0.59 ± 0.12
Nickel (Ni)	<0.01	<0.01	<0.01	<0.01
Lead (Pb)	0.22 ± 0.15	0.19 ± 0.02	0.13 ± 0.04	0.18 ± 0.05
Strontium (Sr)	2.72 ± 0.09	0.19 ± 0.04	0.52 ± 0.05	1.14 ± 1.38
Zinc (Zn)	0.88 ± 0.03	1.23 ± 0.06	0.90 ± 0.02	1.00 ± 0.20

H-1: honey samples from the region of Faisalabad, Punjab; H-2: honey from the region of Kashmir, Pakistan; H-3: honey from the region of Gujranwala, Punjab.

**Table 9:** Pearson correlation of toxic metals in honey samples.

Mineral	Al	Bi	Cd	Cr	Cu	Mn	Ni	Pb	Sr	Zn
Al	1									
Bi	0.713026	1								
Cd	−0.75396	−0.077	1							
Cr	−0.71484	−0.0194	0.998337	1						
Cu	−0.78934	−0.99329	0.191812	0.134924	1					
Mn	0.959199	0.4857	−0.90893	−0.88338	−0.58356	1				
Ni	0.753956	0.076996	−1	−0.99834	−0.19181	0.908928	1			
Pb	0.60913	−0.12172	−0.98025	−0.99002	0.006091	0.808504	0.980245	1		
Sr	0.997496	0.66166	−0.79852	−0.7625	−0.74395	0.976791	0.798523	0.663688	1	
Zn	−0.69951	−0.99982	0.057946	0.000307	0.990897	−0.46892	−0.05795	0.140654	−0.64722	1

**Table 10:** Human health risk assessment of H-1, H-2, and H-3 (mean) honey samples.

Toxic Metals	Average Daily Dose (Children)	Average Daily Dose (Adult)	Total Hazard Quotient (Children)	Total Hazard Quotient (Adult)	Carcinogenic Risk Assessment (Children)	Carcinogenic Risk Assessment (Adult)
Al	6.06 × 10^−5^	6.36 × 10^−6^	6.06 × 10^−5^	6.36 × 10^−6^	8.48 × 10^−5^	8.90 × 10^−6^
Bi	7.19 × 10^−7^	7.55 × 10^−8^	3.60 × 10^−6^	3.77 × 10^−7^	N/A	N/A
Cd	6.00 × 10^−8^	6.30 × 10^−9^	6.00 × 10^−5^	6.30 × 10^−6^	2.28 × 10^−8^	2.39 × 10^−9^
Cr	3.53 × 10^−6^	3.71 × 10^−7^	2.36 × 10^−6^	2.47 × 10^−7^	1.77 × 10^−6^	1.85 × 10^−7^
Cu	2.47 × 10^−6^	2.59 × 10^−7^	6.17 × 10^−5^	6.48 × 10^−6^	4.20 × 10^−6^	4.41 × 10^−7^
Mn	4.78 × 10^−6^	5.02 × 10^−7^	3.41 × 10^−5^	3.58 × 10^−6^	N/A	N/A
Ni	5.34 × 10^−8^	5.60 × 10^−9^	2.67 × 10^−6^	2.80 × 10^−7^	9.07 × 10^−5^	9.52 × 10^−9^
Pb	1.44 × 10^−6^	1.51 × 10^−7^	4.10 × 10^−4^	4.31 × 10^−5^	1.22 × 10^−8^	1.28 × 10^−9^
Sr	1.82 × 10^−5^	1.91 × 10^−6^	3.03 × 10^−5^	3.18 × 10^−6^	N/A	N/A
Zn	5.86 × 10^−6^	6.15 × 10^−7^	1.95 × 10^−5^	2.05 × 10^−6^	N/A	N/A
Hazard Index			Cancer Risk			
HI (children)		HI (adult)	CR (children)		CR (adult)	
6.85 × 10^−4^		7.19 × 10^−5^	9.09 × 10^−5^		9.54 × 10^−6^	
Al	2.75 × 10^−5^	2.89 × 10^−6^	2.75 × 10^−5^	2.89 × 10^−6^	3.85 × 10^−5^	4.05 × 10^−6^
Bi	2.81 × 10^−7^	2.95 × 10^−8^	1.41 × 10^−6^	1.48 × 10^−7^	N/A	N/A
Cd	6.00 × 10^−8^	6.30 × 10^−9^	6.00 × 10^−5^	6.30 × 10^−6^	2.28 × 10^−8^	2.39 × 10^−9^
Cr	3.63 × 10^−6^	3.81 × 10^−7^	2.42 × 10^−6^	2.54 × 10^−7^	1.81 × 10^−6^	1.90 × 10^−7^
Cu	5.96 × 10^−6^	6.25 × 10^−7^	1.49 × 10^−4^	1.56 × 10^−5^	1.01 × 10^−5^	1.06 × 10^−6^
Mn	3.55 × 10^−6^	3.73 × 10^−7^	2.54 × 10^−5^	2.66 × 10^−6^	N/A	N/A
Ni	5.34 × 10^−8^	5.60 × 10^−9^	2.67 × 10^−6^	2.80 × 10^−7^	9.07 × 10^−8^	9.52 × 10^−9^
Pb	1.25 × 10^−6^	1.31 × 10^−7^	3.57 × 10^−4^	3.74 × 10^−5^	1.06 × 10^−8^	1.11 × 10^−9^
Sr	1.24 × 10^−6^	1.30 × 10^−7^	2.07 × 10^−6^	2.17 × 10^−7^	N/A	N/A
Zn	8.18 × 10^−6^	8.59 × 10^−7^	2.73 × 10^−5^	2.86 × 10^−6^	N/A	N/A
Hazard Index			Cancer Risk			
HI (children)		HI (adult)	CR (children)		CR (adult)	
6.54 × 10^−4^		6.87 × 10^−5^	5.06 × 10^−5^		5.31 × 10^−6^	
Al	3.42 × 10^−5^	3.59 × 10^−6^	3.42 × 10^−5^	3.59 × 10^−6^	4.79 × 10^−5^	5.03 × 10^−6^
Bi	6.82 × 10^−7^	7.15 × 10^−8^	3.41 × 10^−6^	3.58 × 10^−7^	N/A	N/A
Cd	6.00 × 10^−8^	6.30 × 10^−9^	6.00 × 10^−5^	6.30 × 10^−6^	2.28 × 10^−8^	2.39 × 10^−5^
Cr	3.75 × 10^−6^	3.93 × 10^−7^	2.50 × 10^−6^	2.62 × 10^−7^	1.87 × 10^−6^	1.97 × 10^−7^
Cu	3.18 × 10^−6^	3.33 × 10^−7^	7.94 × 10^−5^	8.33 × 10^−6^	5.40 × 10^−6^	5.67 × 10^−7^
Mn	3.41 × 10^−6^	3.58 × 10^−7^	2.44 × 10^−5^	2.56 × 10^−6^	N/A	N/A
Ni	5.34 × 10^−8^	5.60 × 10^−9^	2.67 × 10^−6^	2.80 × 10^−7^	9.07 × 10^−8^	9.52 × 10^−9^
Pb	8.58 × 10^−7^	9.00 × 10^−8^	2.45 × 10^−4^	2.57 × 10^−5^	7.29 × 10^−9^	7.65 × 10^−10^
Sr	3.48 × 10^−6^	3.65 × 10^−7^	5.80 × 10^−6^	6.09 × 10^−7^	N/A	N/A
Zn	6.01 × 10^−6^	6.31 × 10^−7^	2.00 × 10^−5^	2.10 × 10^−6^	N/A	N/A
Hazard Index			Cancer Risk			
HI (children)		HI (adult)	CR (children)		CR (adult)	
4.78 × 10^−4^		5.01 × 10^−5^	5.53 × 10^−5^		5.81 × 10^−6^	

N/A, Not Available. Al—aluminum, Bi—bismuth, Cd—cadmium, Cr—chromium, Cu—copper, Mn—manganese, Ni—nickel, Pb—lead, Sr—strontium, and Zn—zinc.

## Data Availability

The original contributions presented in this study are included in the article. Further inquiries can be directed to the corresponding authors.
